# CD40 Ligand Deficient C57BL/6 Mouse Is a Potential Surrogate Model of Human X-Linked Hyper IgM (X-HIGM) Syndrome for Characterizing Immune Responses against Pathogens

**DOI:** 10.1155/2015/679850

**Published:** 2015-05-03

**Authors:** Catalina Lopez-Saucedo, Rodolfo Bernal-Reynaga, Jesus Zayas-Jahuey, Silvia Galindo-Gomez, Mineko Shibayama, Carlos Garcia-Galvez, Sergio Estrada-Parra, Teresa Estrada-Garcia

**Affiliations:** ^1^Department of Molecular Biomedicine, CINVESTAV-IPN, Avenue IPN 2508, Zacatenco, 07360 México, DF, Mexico; ^2^Department of Immunology, Escuela Nacional de Ciencias Biológicas IPN, Prolongación de Carpio y Plan de Ayala, 11340 México, DF, Mexico; ^3^Department of Infectomics and Molecular Pathogenesis, CINVESTAV-IPN, Avenue IPN 2508, Zacatenco, 07360 México, DF, Mexico

## Abstract

Individuals with X-HIGM syndrome fail to express functional CD40 ligand; consequently they cannot mount effective protective antibody responses against pathogenic bacteria. We evaluated, compared, and characterized the humoral immune response of wild type (WT) and C57-CD40L deficient (C57-CD40L^−/−^) mice infected with *Citrobacter rodentium*. Basal serum isotype levels were similar for IgM and IgG3 among mice, while total IgG and IgG2b concentrations were significantly lower in C57-CD40L^−/−^ mice compared with WT. Essentially IgG1 and IgG2c levels were detectable only in WT mice. C57-CD40L^−/−^ animals, orally inoculated with 2 × 10^9^ CFU, presented several clinical manifestations since the second week of infection and eventually died. In contrast at this time point no clinical manifestations were observed among C57-CD40L^−/−^ mice infected with 1 × 10^7^ CFU. Infection was subclinical in WT mice inoculated with either bacterial dose. The serum samples from infected mice (1 × 10^7^ CFU), collected at day 14 after infection, had similar *C. rodentium*-specific IgM titres. Although C57-CD40L^−/−^ animals had lower IgG and IgG2b titres than WT mice, C57-CD40L^−/−^ mice sera displayed complement-mediated bactericidal activity against *C. rodentium*. *C. rodentium*-infected C57-CD40L^−/−^ mice are capable of producing antibodies that are protective. C57-CD40L^−/−^ mouse is a useful surrogate model of X-HIGM syndrome for studying immune responses elicited against pathogens.

## 1. Introduction

The hyper IgM syndromes (HIGM) are a group of primary immune deficiency disorders which are the result of a variety of genetic defects affecting the interaction between T-lymphocytes and B-lymphocytes, including class switch recombination and somatic hypermutation [1]. Among these HIGM syndromes the X-linked HIGM syndrome (X-HIGM) is the most frequently identified accounting for about 65 to 70% of all cases. X-HIGM syndrome results from mutations in the* cd40l* gene that encodes for the CD40 ligand (CD40L) molecule [2]. CD40L is an inducible type II membrane glycoprotein, found on the surface of T cells after antigen stimulation that binds to the CD40 molecule on B cells. CD40-CD40L interaction plays a major role in isotype switching, induction of B and T cell proliferation, B cell affinity maturation, and germinal centre formation [3]. Cases of X-HIGM have been described in industrialized countries [4] and also in less developed areas of the world, as India and Latin America [5–7]. In a study conducted in six Latin American countries, including Mexico, of a total of 58 patients with HIGM clinical features, 37 had genetic defects; of these 35 patients had CD40L deficiencies [6], revealing that X-HIGM is as well the most frequent HIGM syndrome in this region.

X-HIGM patients are characterized by low IgG and IgA serum concentrations and normal or elevated IgM concentrations [1]. In addition, X-HIGM patient's lymph nodes lack germinal centres and their antigen-specific responses may be decreased or are absent [1]. Patients develop clinical symptoms by age one year, and more than 90% are symptomatic by age four years [1, 8]. The range of clinical findings varies, even within the same family, and includes recurrent upper- and lower-respiratory tract bacterial infections, opportunistic infections, and recurrent or protracted diarrhoea [1]. Diarrhoea syndromes occur in over 50% of patients [2].* Cryptosporidium parvum *has been the most common pathogen isolated from faeces of X-HIGM patients with diarrhoea from industrialized countries [1, 2], while it has been reported that* Giardia lamblia* was the most common pathogen identified in X-HIGM patients from Latin America [6]. However, in at least 50% of X-HIGM patients with recurrent or protracted diarrhoea no infectious agent can be detected [8]. This could be due to the fact that not all enteric pathogens are sought out. For instance, diarrheagenic* Escherichia coli* (DEC) are major pathogens associated with both acute and protracted bacterial diarrhoea worldwide, even so* E. coli* strains isolated from diarrhoeal stool samples are still considered commensal flora [9]. Hence, potentially DEC could be an important unknown cause of diarrhoea among X-HIGM patients.

In 1994, two C57BL/6 CD40L-deficient mice (C57-CD40L^−/−^) were developed by two independent groups [10, 11]. As in humans C57-CD40L^−/−^ mice are characterized by low serum concentrations of IgG and IgA but normal, lower, or higher serum concentrations of IgM [10–12]. The C57-CD40L^−/−^ mice have been successfully used to develop infection models of human intestinal pathogens including, for example,* C. parvum*, one of the most common pathogens identified among X-HIGM patients [13], and enterotoxigenic* E. coli*, a DEC pathotype [12].* Citrobacter rodentium* is a natural noninvasive intestinal pathogen of mice that produces deathly diarrhoea in suckling mice and causes transmissible subclinical colonic hyperplasia in adult mice [14, 15]. Furthermore,* C. rodentium* mouse infection model has become the “gold standard” animal model for investigating the virulence mechanisms of pathogens producing the attaching-and-effacing (A/E) lesion [14, 16, 17]. A/E bacteria encompass the human enteric pathogens, enteropathogenic* E. coli* (EPEC) and enterohaemorrhagic* E. coli* (EHEC).* C. rodentium* studies have demonstrated that mice systemic pathogen-specific IgG and CD4^+^ T cell responses are required for survival and resolution of bacteria colonizing the gut epithelium [18–20]. Furthermore, protective serum antibody responses in acute* C. rodentium* infection consisted of pathogen-specific IgM and IgG2b/IgG2c responses; these profiles are consistent with complement-fixing antibodies [20]. Therefore, the aims of this study were to evaluate and compare the oral infection* C. rodentium* in WT and C57-CD40L^−/−^ mice and their systemic antibody response against this pathogen, as well as to establish if C57-CD40L^−/−^ mice are capable of producing complement-fixing antibodies against* C. rodentium*.

## 2. Materials and Methods

### 2.1. Animals

Five- to eight-week-old female and male wild type (WT) C57BL/6 mice and C57-CD40L^−/−^ mice, derived from a C57BL/6 background, were used (Jackson Laboratory Bar Harbor, Maine, USA). Mice came from colonies that were specific-pathogen-free and sentinel animals were screened for common murine pathogens every 6 months or once a year. All animals were housed in autoclaved microisolator filtered cages, with sterile bedding and free access to sterilized food and water. During the experiments the cage beddings were changed daily. All experimental procedures were reviewed and approved by the CINVESTAV-IPN animal Ethical Committee.

### 2.2. Bacterial Strain and Inoculum Preparation

*Citrobacter rodentium* strain DBS 100 was used in all experiments, and this strain was kindly provided by Dr. Jose Luis Puente (Department of Molecular Microbiology, Institute of Biotechnology, UNAM, Mexico).* Citrobacter rodentium* was cultured on MacConkey agar for 18–24 h at 37°C. Briefly, one colony was grown overnight in 5 mL of Luria-Bertani (LB) broth at 37°C without shaking. Next day 1 mL of bacterial culture was resuspended in 50 mL of fresh LB broth, was incubated with shaking at 37°C for additional 4 h, and then was centrifuged at 13,000 rpm and the pellet was washed twice and resuspended in 1 mL of sterile physiological saline (SPS). Bacterial concentration was determined by measuring the optical density (OD) at 600 nm (Smart Spec 3000, Biorad), one OD = 5 × 10^8^ CFU/mL. Finally, the bacterial suspension was adjusted to the concentration required for the experiments in a final volume of 50 *μ*L.

### 2.3. Mice Inoculation

Mice were fasted for 6 h before oral inoculation, via a feeding needle, with* C. rodentium*. The inoculation dose was verified retrospectively by plating serial dilutions on trypticase soy agar plates and enumerating colonies. Sham controls were administered with an equal volume of SPS (50 *μ*L).

### 2.4. Monitoring of Faecal Shedding of* Citrobacter rodentium*

To determine bacterial numbers in the stools, faecal pellets were collected from individual mice, weighed, and homogenised in 1 mL of SPS. Bacterial homogenates serial dilutions were plated on MacConkey agar and colony-forming units (CFU) were determined after overnight incubation at 37°C.* C. rodentium* colonies (pink-red centre with a transparent rim, slightly translucent) were selected and their identity was confirmed by a specific intimin-B protein gene (*eaeB*) PCR developed by us. We prepared bacterial lysates by resuspending single colonies in 1 mL of deionized water (Milli-Q System, Millipore, Bedford, MA), boiled for 1 min, and then freezing them until needed. Based on the* C. rodentium* intimin B gene sequence (GenBank accession number AF311901) the following primers were designed: 5′-tgagcgcccggcaaatggtt-3′ (forward) and 5′-tgtgcgctttggcttccgct-3′ (reverse). Briefly 1 *μ*L of bacterial lysate and primers in a final concentration of 0.2 *μ*M were added to a PCR tube that contained 24 *μ*L of reaction mix whose concentration has been previously described [21] and subjected to the following cycling conditions: 50°C (2 min, 1 cycle); 95°C (5 min, 1 cycle); 95°C, 50°C, and 72°C (45 s each temperature, 40 cycles) and a final extension step (10 min, 72°C) in a thermal cycler (iCycler System, Bio-Rad Laboratories, Inc., Hercules, CA). A 555-bp PCR product was visualized by agarose gel electrophoresis and ethidium bromide staining.

### 2.5. Histological Analysis

Mice were sacrificed by cardiac exsanguination under chloroform effect at day 14 after inoculation and the colons were removed. Segments (1 cm) of terminal colon from each mouse were collected, longitudinally cut, and pinned out flat with the mucosal side up. The tissues were fixed in 4% paraformaldehyde in PBS for 48 h at room temperature, dehydrated by gradient ethanol, cleared by xylene, and embedded in paraffin. Sections of 5 *μ*m were prepared and stained with haematoxylin and eosin. Sections were evaluated for changes in the mucosal architecture and the presence of an inflammatory infiltrate. Villus high was measured on ten villi in each slide, three slides per mice strain.

### 2.6. *Citrobacter rodentium* Whole-Cell Sonicate Preparation

The bacterium was grown as described above (see bacteria strain and inoculum preparation section). The bacteria culture was centrifuged at 13,000 rpm and the pellet was washed twice with phosphate-buffered saline pH 7.4 (PBS). Then the bacterial pellet was resuspended in 5 mL of PBS and subsequently sonicated five times by periods of 1 min, pulse each 10 s, and 40 *μ*m wave amplitude (ultrasonic processor). The bacterial sonicate was centrifuged at 13,000 rpm for 10 min and the supernatant was used for ELISA assays. Protein concentration was assessed by Bradford test (Bio-Rad 500-0006).

### 2.7. Determination of Total Basal Immunoglobulin Concentrations

Mice blood samples were collected from the mice tail vein and centrifuged, and serum aliquots were frozen at −70°C until tested. Total basal IgM, IgG, and IgG subclasses and IgA concentrations were determined in the serum by comparing the values of test sample dilution series in ELISA, with isotype-specific control standard curves (Cappel 50335, Jackson Immunoresearch 015-000-003, and Cappel 50325 for the IgM and IgG-IgG subclasses and IgA, resp.). Briefly, individual wells of flat bottom ELISA plates (Corning Inc., Costar 3590) were coated with 60 *μ*L of capture antibody for IgM (Jackson Immunoresearch 715 005-140), for IgG and IgG subclasses (ZYMED 61-6400) and IgA (Southern Biotech 1165-01), in a final concentration of 1.0 *μ*g/mL in carbonate-bicarbonate buffer pH 9.6 and incubated overnight at 4°C. Then plates were washed with 0.1% v/v Tween 20 in phosphate-buffered saline (PBS-T) and subsequently blocked with 1% BSA in PBS-T (blocking solution) for 1 h at 37°C. Then 60 *μ*L of serum samples diluted in blocking solution was added in duplicate into wells for 1 h at 37°C. Serum samples were diluted according to the Ig to be evaluated (see Table 1). Followed by washing and incubation with respective anti-mouse Ig-horseradish peroxidase conjugated secondary antibodies diluted 1 : 1000 in blocking solution for IgM (Pierce, 31440), IgG (Invitrogen, G21040), and IgA (Sigma A 4789) and 1 : 4000 for IgG subclasses (IgG1, IgG2b, IgG2c, and IgG3, SouthernBiotech, 1070-05, 1090-05, 1079-05, and 1100-05, resp.). All assays were developed using ABTS peroxidase substrate system (Sigma, A1888) and plates were read at 405 nm on an ELISA reader (Tecan, Sunrise). OD shown by the background controls was subtracted from the OD of each test sample.

### 2.8. *C. rodentium*-Specific Antibody Titres

At postinoculation selected times mice blood samples were collected from the mice tail vein and centrifuged and serum aliquots were frozen at −70°C until tested. Microtiter plates were coated overnight at 4°C with 60 *μ*L of carbonate-bicarbonate buffer pH 9.6 containing whole-cell sonicate lysate (10 *μ*g/mL). The plates were washed with PBS-T and blocked with blocking solution 1 h, 37°C. Then serially diluted serum in blocking solution was added in duplicate and incubated 1 h at 37°C. The next steps were developed as described above.

### 2.9. Western Blot Analysis

For immunoblots,* C. rodentium *whole-cell sonicate (35 *μ*g per well) boiled and unboiled was resolved in 10% SDS-PAGE gels (90 V, 400 mA for 110 min) and transferred to nitrocellulose membranes (90 V, 400 mA for 2 h). The membranes were blocked with PBS Tween 20 0.5%, plus BSA (10 mg/mL), dextrose 1 M, and 10% (v/v) of glycerol (blocking solution) overnight at 4°C by slow shaking. Membranes were incubated with serum diluted 1 : 200 overnight at 4°C. After incubation, membranes were washed (10 min per wash) in PBS-T 0.1% three times and three times with PBS. Then membranes were incubated for 2 h at room temperature with anti-mouse Ig-horseradish peroxidase conjugated secondary antibodies for IgM (Pierce, 31440) and IgG (Invitrogen, G21040) diluted 1 : 1000 in blocking solution. After this period the membranes were washed once more as previously described. HRP-bound antibody was developed with Western Blotting Luminol Reagent (Santa Cruz) and visualized using a ChemiDocTM MP system (Bio-Rad).

### 2.10. Complement-Dependent Bactericidal Antibody Assays

Mice serum to be tested was heat-inactivated by incubation at 56°C for 30 min and was diluted in Hanks' solution. WT mouse serum was first diluted in 1 : 100, followed by a 1 : 500 dilution and then twofold dilutions until 1 : 16000. C57-CD40L^−/−^ mouse serum was first diluted 1 : 50, followed by twofold dilutions until 1 : 1600. Then 30 *μ*L of each dilution was added to a tube containing 20 *μ*L of a bacterial suspension (1000 ± 10) and incubated at 37°C for 30 min. After this period 50 *μ*L of exogenous complement (guinea pig serum 10% in Hanks' solution) was added to each tube and incubated at 37°C for 30 min. Both incubations were done in a shaker at 25 rpm. To each tube 900 *μ*L of Hanks' solution was added and then diluted 1 : 10, and 100 *μ*L was taken and plated on trypticase soy agar plates, in duplicate, and incubated overnight at 37°C. Next day the number of CFU per plate was determined. The serum bactericidal antibody titre was defined as the reciprocal of the highest serum dilution that produced ≥50% killing in relation to the killing observed for the negative control (viable-bacteria count control). In order to validate the bactericidal activity four negative controls were included in all assays: (1) viable-bacteria count control (bacteria suspension without serum or guinea pig complement), (2) inactivated serum control (inactivated serum and bacteria suspension without guinea pig complement), (3) guinea pig complement control (guinea pig complement and bacteria suspension without serum), and (4) inactivated guinea pig complement control (inactivated guinea pig complement, inactivated serum, and bacteria suspension).

### 2.11. Statistical Analysis

Kolmogorov-Smirnov test was used to determine the data distribution. The paired Student's *t*-test was used to compare normally distributed values from groups of mice. The nonparametric Mann-Whitney test was used to compare nonnormally distributed values. Differences with a *P* value < 0.05 were considered significant. GraphPad Prism software (version 5.0) was used to generate graphs and to analyse the data.

## 3. Results

### 3.1. *Citrobacter rodentium* Course of Infection in Wild Type and C57-CD40L^−/−^ Mice

**Figure 1:**
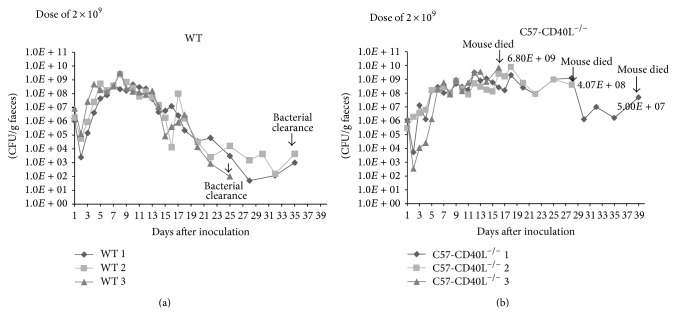
Course of* C. rodentium* infection in wild type (WT) and C57-CD40L^−/−^ mice. (a) WT mice (black lines) and (b) C57-CD40L^−/−^ mice (grey lines) were orally inoculated with 2 × 10^9^ CFU of* C. rodentium *and faecal bacterial numbers were determined. CFU/g of faeces per day and per mouse was plotted. While WT mice infected with 2 × 10^9^ CFU of* C. rodentium* cleared bacterial infection, C57-CD40L^−/−^ mice died.

In WT mice* C. rodentium* infection has been well characterized but has not been described in C57-CD40L^−/−^ mice. Therefore, a set of mice of each strain was orally inoculated with a dose of 2 × 10^9^ CFU of* C. rodentium *(a dose that already has been standardized for this model of infection). Mice were followed up until day 39; stools were collected daily for 18 days and every other day until day 39. CFU per gram of faeces (CFU/g) were similar in both mice strains during the first week of infection, though C57-CD40L^−/−^ faeces were softer compared with WT faeces (Figures 1(a) and 1(b)). During the second week of infection once more both mice strains had similar CFU/g; even so, C57-CD40L^−/−^ mice presented several clinical manifestations such as weight loss, dehydration, coat ruffling, hunched posture, and listlessness. As shown in Figure 1(b), all three C57-CD40L^−/−^ mice died (the first one at day 17, the second at day 29, and the last one at day 39). WT mice only produced soft faeces at week two but did not present any other clinical manifestation during the entire experiment.

### 3.2. *Citrobacter rodentium* Infection in WT and C57-CD40L^−/−^ Mice with a Dose of 5 × 10^8^ and 1 × 10^7^

**Figure 2:**
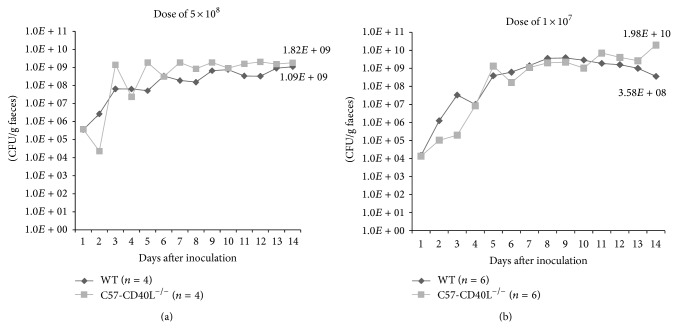
*C. rodentium *infection in WT and C57-CD40L^−/−^ mice. WT (black lines) and C57-CD40L^−/−^ mice (grey lines) were orally inoculated with 5 × 10^8^ (a) and 1 × 10^7^ (b) CFU of* C. rodentium*. Mean CFU/g of faeces per day and per mice strain was plotted. WT = wild type. No significant differences were observed between CFU per mice strain.

In order to minimize the observed clinical manifestations induced by a dose 2 × 10^9^ CFU in C57-CD40L^−/−^ two lower* C. rodentium* infectious doses were tested. Both mice strains were orally inoculated with 5 × 10^8^ CFU and followed up for 14 days. Stools were collected daily and both strains had similar numbers of CFU/g (Figure 2(a)). As with the dose of 2 × 10^9^ CFU C57-CD40L^−/−^ mice produced soft faeces during the first week and once more during the second week of infection several clinical manifestations were observed. Therefore, a dose of 1 × 10^7^ CFU was tested, animal were followed up for 14 days, and it was observed that* C. rodentium* CFU/g among mice strains was not significantly different (Figure 2(b)). Even though, during the first week of infection, all C57-CD40L^−/−^ mice had soft faeces, none presented any other clinical manifestations during the second week of infection. As with the dose of 2 × 10^9^, infection was subclinical in WT mice inoculated with either bacterial dose.

### 3.3. Morphological and Histological Evaluation of Colons at 14 Days after Infection

**Figure 3:**
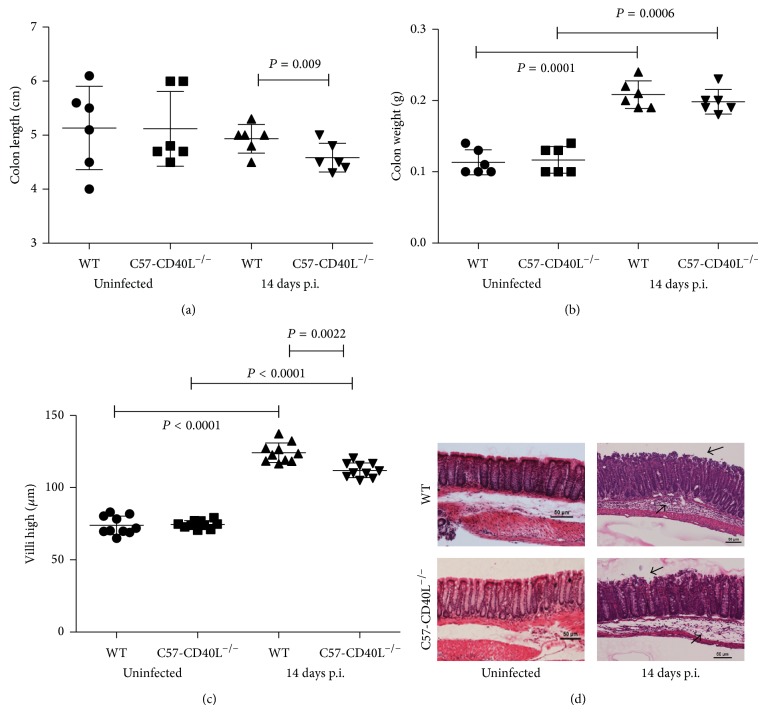
Morphological and histological analysis. Colons of wild type (WT) (*n* = 6) and C57-CD40L^−/−^  (*n* = 6) mice uninfected and orally inoculated with 1 × 10^7^ CFU of* C. rodentium *were removed at day 14 after inoculation (p.i.). (a) Colon length. Infected C57-CD40L^−/−^ mice colons lengths were significantly shorter than colons of infected WT mice. (b) Colon weight. Colons of infected-WT and -C57-CD40L^−/−^ mice weighted more compared with colons of uninfected mice. (c) Villi high. Intestinal villi of both infected mice strains were significantly higher than villi of uninfected mice. Villi of infected WT mice were higher compared with villi of infected C57-CD40L^−/−^ mice. Mean comparisons were done by paired Student's *t*-test. (d) Hematoxylin and eosin stained sections of distal colons. Arrows denote submucosal inflammatory cellular infiltrates and epithelial damage in the mucosa of infected mice.

A hallmark feature of* C. rodentium* infection is colonic hyperplasia measured as an increase in crypt and villus high that is maximal after 10–14 days after infection [15, 22]. Other features of inflammation due to* C. rodentium* infection are decreased in colon length and increase in colon weight [23]. In order to establish if there were morphological and histological differences among mice strains after* C. rodentium *infection with 1 × 10^7^ CFU, both mice strains were inoculated with a dose 1 × 10^7^ CFU and control mice with sterile physiological saline (SPS). Then at day 14 after infection all mice were sacrificed and colons were removed. Colons from infected C57-CD40L^−/−^ mice were shorter (*P* = 0.009) compared with colons of infected WT mice (Figure 3(a)). Difference in colon weight and villi high was pronounced between* C. rodentium*-infected mice (*P* < 0.001) and uninfected mice (Figures 3(b) and 3(c)). On the other hand, villi of infected WT were significantly higher (*P* = 0.0022) than in C57-CD40L^−/−^ mice (Figure 3(c)). Histological analysis of colonic tissue of infected animals showed a mixed cellular infiltrate mostly in the submucosa but also some in the lamina propria. Surface epithelial erosion although present in both mice tissues was slightly more evident in C57-CD40L^−/−^ mice (Figure 3(d)).

### 3.4. Basal Total Concentrations of Serum Immunoglobulins

**Figure 4:**
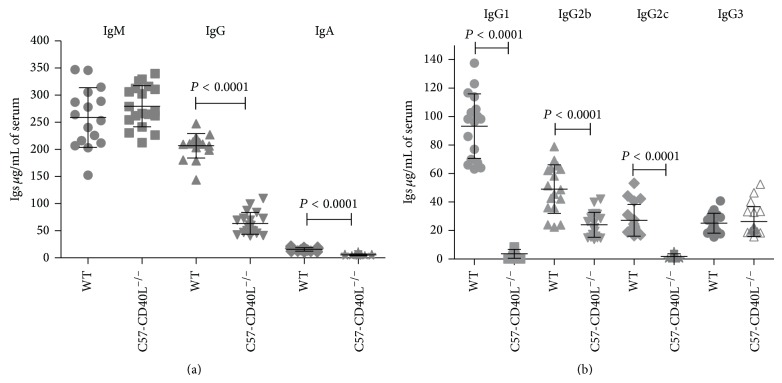
Basal Ig isotypes sera concentrations of WT (*n* = 17) and C57-CD40L^−/−^  (*n* = 19) mice. (a) Basal IgM, IgG, and IgA concentrations. Ig concentrations were determined by extrapolation from a standard curve. The total mean IgM concentration was similar among mice strains. Total IgG and IgA serum concentrations of C57-CD40L^−/−^ were significantly lower than concentrations of WT mice. (b) Basal IgG subclasses sera concentrations. IgG3 levels were essentially identical between the two groups and all other subclasses concentrations were significantly higher in WT than in CD40L deficient mice. Mean comparisons were done by Mann-Whitney *U*-test. WT = wild type.

Serum was collected from 17 WT and 19 C57-CD40L^−/−^ adult mice (5- to 7-week-old), and isotype concentrations were determined by ELISA. IgM and IgG3 levels were essentially identical between mice strains (Figures 4(a) and 4(b)). Serum concentrations of IgA, total IgG, IgG1, 1gG2b, and IgG2c were significantly lower (*P* < 0.0001) in CD40L deficient mice compared with isotype concentrations of WT (Figures 4(a) and 4(b)). The overall isotype concentration decrease between WT and C57-CD40L^−/−^ mice was as follows: 51% for IgG2b, 65% for IgA, 69% for total IgG, 93% for IgG2c, and 96% for IgG1.

### 3.5. *C. rodentium*-Specific Serum Antibodies

**Figure 5:**
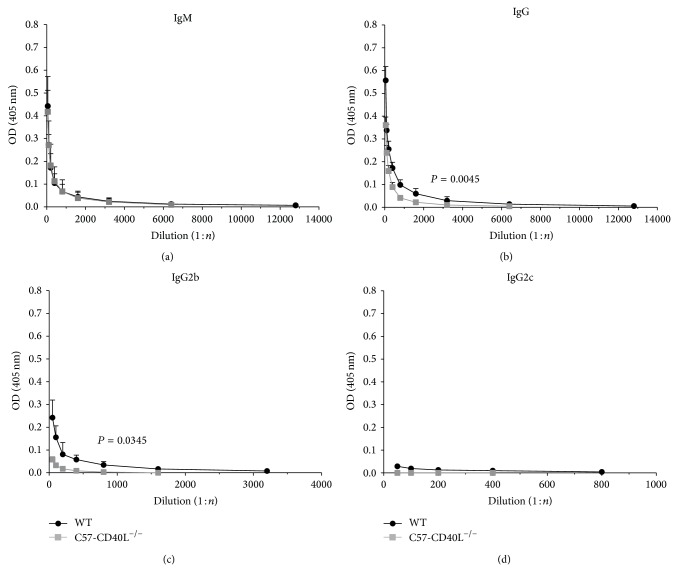
*C. rodentium*-specific sera antibodies of wild type (WT) (*n* = 6) and C57-CD40L^−/−^  (*n* = 6) mice. Mice orally inoculated with 1 × 10^7^ CFU of* C. rodentium *were bled at day 14 after inoculation. Each point of these curves represents the mean ± SD of the OD determinations. (a) The specific IgM titres were essentially identical between the two groups. ((b) and (c)) The specific IgG and IgG2b titres were significantly lower for C57-CD40L^−/−^ mice than WT mice. (d) IgG2c specific antibodies of both WT and C57-CD40L^−/−^ mice were just above baseline and IgG1 and IgG3 isotypes were undetectable. Antibody titres are presented as means and data was analysed by Mann-Whitney *U*-test. OD = optical density, (1 : *n*) = dilution factor.

*C. rodentium* infected WT mice characteristically develop strong pathogen-specific serum IgM responses that peak approximately 2 weeks after oral inoculation with this pathogen, while IgG responses rise above baseline and peak over subsequent weeks [24]. The serum samples of mice inoculated with 1 × 10^7^ CFU were collected at day 14 after inoculation and tested by ELISA using* C. rodentium *whole-cell sonicate as antigen. IgM-titre against* C. rodentium* was similar in both mice strains (Figure 5(a)). WT-infected mice produced significant higher concentrations of specific* C. rodentium* total IgG (*P* = 0.0045) and IgG2b (*P* = 0.0345) antibodies than CD40L deficient-infected mice (Figures 5(b) and 5(c)). At this time point, IgG2c* C. rodentium* specific antibodies were produced just above baseline by WT mice and were undetectable in C57-CD40L^−/−^ mice (Figure 5(d)), and IgG1 and IgG3 anti-*C. rodentium* antibodies were undetectable in both mice strains.

### 3.6. Complement-Mediated Bactericidal Activity of Mice Serum against* C. rodentium*

**Figure 6:**
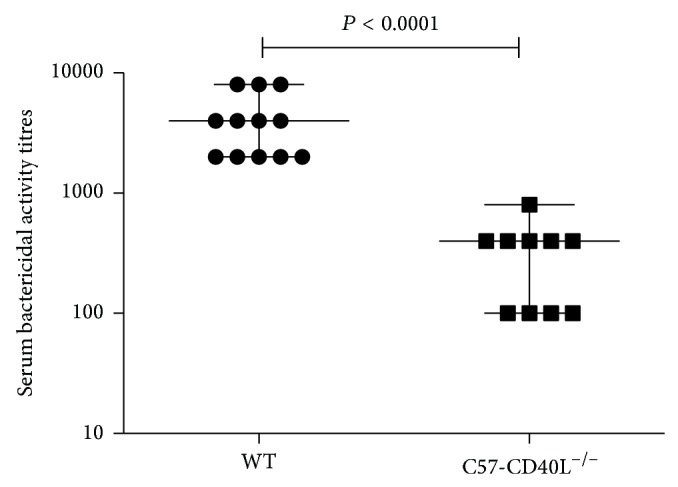
Complement-mediated bactericidal activity. Mice were orally inoculated with 1 × 10^7^ *C. rodentium* CFU and were bled at 14 days after inoculation. Both mice sera had bactericidal activity; C57-CD40L^−/−^ mice (*n* = 11) sera had significantly lower bactericidal activity compared with wild type (WT) (*n* = 12) mice sera. Data for bactericidal activity are presented as medians and data was analysed by Mann-Whitney *U*-test.

The protective serum antibody response in acute* C. rodentium* infection in WT mice consists of complement-fixing IgM and IgG2b/IgG2c antibodies [20]. Therefore, a serum bactericidal assay to measure antibody-dependent complement-mediated killing was implemented. Mice inoculated with a 1 × 10^7^ CFU were bled at day 14 after inoculation. The sera of 11 immune C57-CD40L^−/−^ mice and 12 WT mice were tested in the bactericidal assay. All tested serum samples had bactericidal activity with the exception of a serum from a CD40L deficient mouse. As shown in Figure 6, WT serum bactericidal titres were significantly higher (*P* < 0.0001) compared with bactericidal titres of C57-CD40L^−/−^ mice.

### 3.7. Western Blots

**Figure 7:**
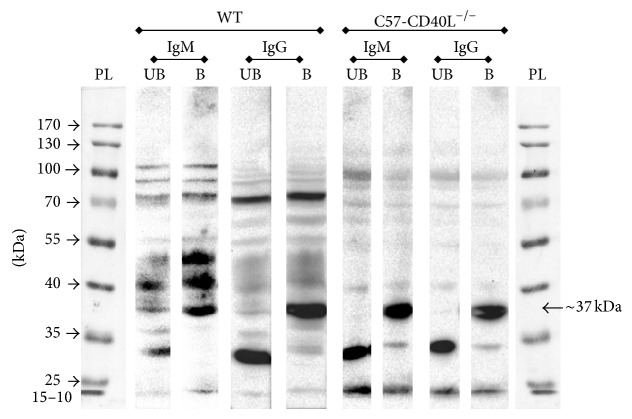
Western blot analysis of* C. rodentium *antiserum of wild type mice (WT) and C57-CD40L^−/−^ mice. Mice were orally inoculated with 1 × 10^7^ *C. rodentium* CFU and were bled at 14 days after inoculation. Whole cell lysates were resolved in 10% gels by SDS-PAGE, and the serum was diluted 1 : 200. PL = protein ladder, UB = unboiled lysate, and B = boiled lysate.

In order to identify specific antigens of* C. rodentium* whole-cell sonicate and serum from three individual mice per group were probed by Western blotting.  Figure 7 shows a representative Western blot for each serum mice strain revealing that both C57-CD40L^−/−^ and WT mouse reacted against various antigens. However, the genesis of certain isotypes against* C. rodentium* antigens was clearly CD40L dependent (Figure 7). For instance, when boiled sonicates were used as an antigen in immunoblot assays, IgM antibodies from infected WT mice recognized predominantly three bands (~50 kDa, ~42 kDa, and ~37 kDa) while only one band (~37 kDa band) of the three was recognized by the IgM antibodies of infected CD40L deficient mice. Moreover, it seems that CD40L-independent IgM and IgG antibodies are predominately directed towards the same antigens present in unboiled and boiled* C. rodentium* sonicates, compared with CD40L-dependent IgM and IgG antibodies (Figure 7).

## 4. Discussion

Individuals with X-HIGM syndrome fail to express functional CD40L and as a consequence they cannot mount an effective protective antibody response to opportunistic bacterial infections. During the nineties, two independent groups, developed C57-CD40L deficient mice and their humoral immune responses, were characterized [10, 11]. Over 50% of X-HIGM patients had recurrent or protracted diarrhoea. However, little is known of the systemic humoral immune response induced in X-HIGM patients against intestinal bacterial pathogens. In the present study we have characterized the humoral immune response of C57-CD40L^−/−^ mice before and after* C. rodentium* infection.* C. rodentium* is a mouse noninvasive pathogen that produces diarrhoea and causes transmissible colonic hyperplasia in suckling and adult mice, respectively [15].

Infection of WT adult mice with a dose of 2 × 10^9^ *C. rodentium* CFU has been shown to be a subclinical self-limiting infection that produces sterilizing immunity.* C. rodentium* first colonizes the caecum, followed by a peak of bacteria load around days 7–10 after infection and complete clearance of the pathogen in the stools occurs 6 weeks after infection [22]. In this study when C57-CD40L^−/−^ mice were orally inoculated with 2 × 10^9^ CFU, several clinical manifestations were observed (weight loss, dehydration, coat ruffling, hunched posture, and listlessness) since the second week of infection and eventually died. It has been shown that* C. rodentium*-specific IgG and CD4^+^ T cell responses are required for WT mice survival and resolution of bacteria colonizing the gut epithelium [18–20]. Therefore, our results suggest that* C. rodentium*-infected C57-CD40L^−/−^ mice do not produce pathogen-specific T cell-dependent antibodies. So far it has been shown that CD40L deficient mice do not produce specific IgG antibodies against TNP-KLH, a T cell-dependent antigen [10, 11]. In agreement, in this study, basal serum T-dependent IgGI and IgG2c isotypes were almost undetectable in C57-CD40L^−/−^ mice whereas T-independent Ig2b levels were only 49% less than that in WT mice. Overall these results are in accordance with previous reports of basal serum isotype levels in C57-CD40L^−/−^ [10–12].

As expected immunoblots revealed that infected-WT mice serum recognized more antigens compared with infected-C57-CD40L^−/−^ serum. Interestingly, immunoblot analysis revealed that CD40L-independent IgM and IgG antibodies are predominately directed towards the same antigens present in unboiled and boiled* C. rodentium* sonicates. Thus CD40L deficient mice are capable of generating* C. rodentium* IgG specific antibodies independent of CD40-CD40L interactions and these antibodies are towards the same antigens recognized by IgM. In the present study a ~37 kDa protein was strongly recognized by* C. rodentium*-infected CD40 deficient mice sera (Figure 7). We speculate that maybe this ~37 kDa protein could be EspB, a 37 KDa protein secreted by pathogens producing A/E lesion as* C. rodentium*, EPEC, and EHEC [25, 26]. EspB has been shown to play an important role in adherence, pore formation, and effector translocation during infection [26]. EspB has also been reported to interact with EspA and EspD [26] simultaneously, and the complexes formed by these three proteins participate in the initial step of bacterial adherence [26]. Nevertheless, the chemical and exact identity of the major* C. rodentium* antigens that were differentially recognized by wild type and CD40L deficient mice sera remains to be determined.

Antibody-mediated immune responses play a critical role in the defence against extracellular pathogens. Hence, if specific antibodies against* C. rodentium* are produced, it is necessary to determine an infectious dose that minimizes the observed clinical manifestations induced by* C. rodentium* in C57-CD40L^−/−^ mice. In the present study, no clinical manifestations were observed among C57-CD40L^−/−^ mice, infected with 1 × 10^7^ CFU, at the second week after inoculation. It has been shown that* C. rodentium*-infected WT mice characteristically develop strong pathogen-specific serum IgM responses that peak approximately at two weeks after infection [24]. Accordingly at this time point we observed that C57-CD40L^−/−^ infected mice mounted a similar serum IgM specific response compared to infected WT mice. Likewise, at day 14 after inoculation CD40L deficient mice had similar* Borrelia burgdorferi*-specific serum IgM antibodies compared to control mice [27]. The importance of IgM in the resolution of spirochaetaemia has been substantiated; for example, mice that are incapable of secreting IgM failed to clear the infection and mechanism as IgM-dependent bacteria phagocytosis has been demonstrated [28]. On the other hand, mice incapable of secreting IgM infected with* C. rodentium *(5 × 10^8^) successfully cleared* C. rodentium* infection [29]. We observed that the major serum IgG subclass produced by* C. rodentium*-infected CD40L deficient mice was the complement fixing IgG2b antibody, though being in lower concentration than WT mice. Also IgG2b is the most prevalent isotype produced in serum and faeces of* C. rodentium-*infected WT mice at 15 days after infection [20]. Several studies have shown* C. rodentium*-specific IgG antibodies are required for WT mice survival and resolution of bacteria colonizing the gut epithelium [20, 29].

It is well known that protective antibody response against* C. rodentium* infection consists largely of complement-fixing antibodies [20]. Therefore, we tested the serum from* C. rodentium*-infected mice with a dose of 1 × 10^7^, from the 14th day after infection, in a complement-mediated bactericidal assay. The serum from all animals (except from the serum of a CD40L deficient mouse) displayed a complement-mediated bactericidal activity against* C. rodentium*. Even so, C57-CD40L^−/−^ mice serum bactericidal antibody titres were significantly lower than titres of WT. To the best of our knowledge, this is the first study that shows that serum from CD40L deficient mice has a complement-mediated bactericidal activity. It remains to be determined if this bactericidal activity provides a survival advantage to* C. rodentium* infected CD40L deficient mice. It has been shown that acute-phase serum transfer from infected C57-CD40L^−/−^ mice with a high dose of* C. rodentium* (5 × 10^8^ CFU) provided a survival advantage of some days to* C. rodentium-*infected CD4 deficient mice recipients but failed to provide complete protection [24]. On the other hand, transfer of serum from* B. burgdorferi*-infected C57-CD40L^−/−^ mouse prevented* B. burgdorferi* infection in a severe combined immunodeficient mouse [27]. Overall, our results suggest that T cells defects due to the albescence of CD40L, which are important in controlling intracellular pathogens, do not play a major role in the clearance of* C. rodentium* and* B. burgdorferi* primary considered to be extracellular pathogens [28].

Likewise serum pathogen-specific IgM and IgG antibodies may have an important role in bacterial clearance in X-HIGM patients. Accordingly, a serum, containing high levels of IgM, from X-HIGM patient infected with* Helicobacter pylori* displayed a 100% killing activity, when tested in a complement-mediated bactericidal assay [30]. It has been documented that sera from X-HIGM patients in addition to normal or elevated concentrations of IgM also contain IgG and IgA in low concentrations, but IgG3 levels are almost normal [31]. IgG3 and IgM isotypes are the more efficient complement-fixing human antibodies [28]. Therefore, serum from XIGH patients may exhibit a complement-mediated bactericidal activity that could result in extracellular bacterial clearance and disease resolving.

## 5. Conclusions

This study demonstrated that C57-CD40L^−/−^ mouse is a useful surrogate model of X-HIGM syndrome.

Since most infections in X-HIGM patients are chronic, C57-CD40L^−/−^ mice will help to study the immune response elicited against human or surrogate human pathogens and to implement treatments that hopefully will help pathogen clearance in X-HIGM patients.

## Figures and Tables

**Table 1:** Serum dilutions to determine basal immunoglobulin concentrations.

Immunoglobulin	Serum dilutions	
	WT	C57-CD40L^−/−^
IgA	1 : 500 and 1 : 1000	1 : 250 and 1 : 500
IgM and total IgG	1 : 4000 and 1 : 8000	1 : 4000 and 1 : 8000
IgG1	1 : 1000 and 1 : 2000	1 : 250 and 1 : 500
IgG2c	1 : 250 and 1 : 500	1 : 250 and 1 : 500
IgG2b	1 : 2000 and 1 : 4000	1 : 1000 and 1 : 2000
IgG3	1 : 1500 and 1 : 3000	1 : 1500 and 1 : 3000

WT = wild type mice.
